# Time‐calibrated phylogeny and ecological niche models indicate Pliocene aridification drove intraspecific diversification of brushtail possums in Australia

**DOI:** 10.1002/ece3.9633

**Published:** 2022-12-15

**Authors:** David Carmelet‐Rescan, Mary Morgan‐Richards, Nimeshika Pattabiraman, Steven A. Trewick

**Affiliations:** ^1^ Wildlife and Ecology, School of Natural Sciences Massey University Palmerston North New Zealand

**Keywords:** Australia, climate cycles, environmental niche modeling, marsupials, molecular dating, possums, subspecies

## Abstract

Major aridification events in Australia during the Pliocene may have had significant impact on the distribution and structure of widespread species. To explore the potential impact of Pliocene and Pleistocene climate oscillations, we estimated the timing of population fragmentation and past connectivity of the currently isolated but morphologically similar subspecies of the widespread brushtail possum (*Trichosurus vulpecula*). We use ecological niche modeling (ENM) with the current fragmented distribution of brushtail possums to estimate the environmental envelope of this marsupial. We projected the ENM on models of past climatic conditions in Australia to infer the potential distribution of brushtail possums over 6 million years. D‐loop haplotypes were used to describe population structure. From shotgun sequencing, we assembled whole mitochondrial DNA genomes and estimated the timing of intraspecific divergence. Our projections of ENMs suggest current possum populations were unlikely to have been in contact during the Pleistocene. Although lowered sea level during glacial periods enabled connection with habitat in Tasmania, climate fluctuation during this time would not have facilitated gene flow over much of Australia. The most recent common ancestor of sampled intraspecific diversity dates to the early Pliocene when continental aridification caused significant changes to Australian ecology and *Trichosurus vulpecula* distribution was likely fragmented. Phylogenetic analysis revealed that the subspecies *T. v. hypoleucus* (koomal; southwest), *T. v. arnhemensis* (langkurr; north), and *T. v. vulpecula* (bilda; southeast) correspond to distinct mitochondrial lineages. Despite little phenotypic differentiation, *Trichosurus vulpecula* populations probably experienced little gene flow with one another since the Pliocene, supporting the recognition of several subspecies and explaining their adaptations to the regional plant assemblages on which they feed.

## Graphical abstract

We use ecological niche modeling (ENM) with the current fragmented distribution of brushtail possums (*Trichosurus vulpecula*) to estimate the current and past environmental envelope of this marsupial. We use whole mitochondrial phylogeny to estimate the timing of intraspecific divergence. The most recent common ancestor of sampled intraspecific diversity dates to the early Pliocene when continental aridification caused significant changes to Australian ecology and *Trichosurus vulpecula* distribution was likely fragmented.

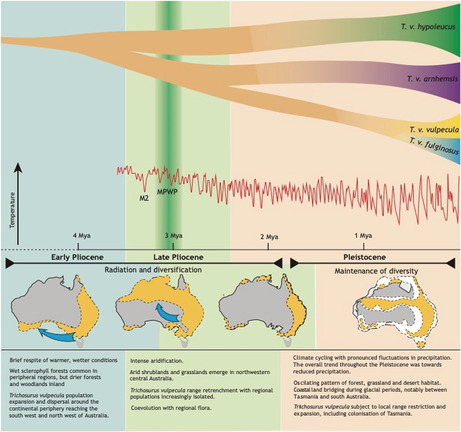

## 1 BACKGROUND

Genetic signatures of range expansion, gene flow, and former barriers provide information that can help our understanding of past and future biological response to environmental change. Phylogeographic studies of Australian animals reveal several different responses to past climatic oscillations. Some studies show that similar to the northern hemisphere, Pleistocene climate cycles were major drivers of species' phylogeographical patterns (Bryant & Fuller, 2014; Strasburg et al., 2007). But the majority of phylogeographic studies of Australian taxa suggest that older climate fluctuations explain current genetic structure (Kuch et al., 2005; Oliver et al., 2010, 2017), especially among marsupials (Macqueen et al., 2010; Potter et al., 2012; Rowe et al., 2008). A rich fossil record of Australian megafauna allows calibration of changes in faunal composition (Hocknull et al., 2020; Miller et al., 2005; Roberts et al., 2001; Saltré et al., 2016; Turney et al., 2008) and it has been suggested that natural climate cycling during the Pleistocene had little impact on mammal assemblages (Prideaux et al., 2007). To determine whether current patterns of genetic structure within a single species date to events that occurred more than 2 million years ago, we examined the phylogeography of the Australian brushtail possum *Trichosurus vulpecula*.

**FIGURE 1:**
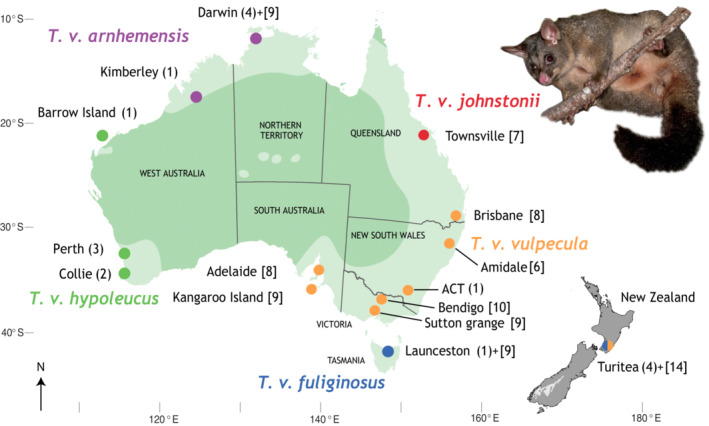
Sampling locations in Australia and New Zealand of *Trichosurus vulpecula* brushtail possums used for phylogenetic analyses. Spot colors coded by putative subspecies identification. The number of samples providing mitogenome sequences for molecular dating (in parentheses) and number of samples providing D‐loop sequence [in square brackets]. In lighter green is the putative current distribution of brushtail possum in Australia (Kerle, 2002). Gray brushtail possum inset credit: Tony Jewell.

Among Australia's endemic marsupial fauna, the brushtail possum *Trichosurus vulpecula* is one of the most widespread with a geographic range spanning the continent of nearly 7.7 million km^2^. Populations of brushtail possum exist at both the east and west extremities of Australia, some 4000 km apart, and the species spans the temperate south to the tropical north (Figure 1). Brushtail possums are arboreal herbivores, and across their geographic range, they interact with regional floras in ecologically distinct regions such as Jarrah and Kerri eucalyptus forest in Southwest Australia, tropical rainforests of Queensland, and temperate Tasmanian vegetation (Kerle, 1984). Despite this wide geographic and ecological range, the existence of numerous Aboriginal names (Abbott, 2012), and subspecies names for geographic races, no reliable diagnostic traits distinguishing brushtail possum populations are apparent from skull morphometrics, allozymes, or chromosomes (Kerle et al., 1991). The six subspecies of *Trichosurus vulpecula* are recognized based on geographic location, size, and fur color (How & Kerle, 1995), and show considerable physiological variation across Australia (Cooper et al., 2018). Brushtail possum populations differ in their exposure to plant chemical defenses as they forage on plants known to contain compounds toxic to mammals including *Erythrophleum*, *Acacia*, *Eucalyptus*, and *Gastrolabium*, and as a result, have evolved different tolerances to toxins. For example, brushtail possums in Southwest Australia (koomal) have an LD50 160 times higher than their cousins (bilda) in East Australia (Twigg et al., 1996) when exposed to potent toxin fluoroacetate (known as 1080; Leong et al., 2017). This emergence of physiological adaptations specific to regional flora demonstrates the coevolutionary interaction resulting in genomic divergence of spatial populations (Mead et al., 1985; Oliver & King, 1979), which implies a sustained interaction counter to the impression drawn from morphology.

**FIGURE 2:**
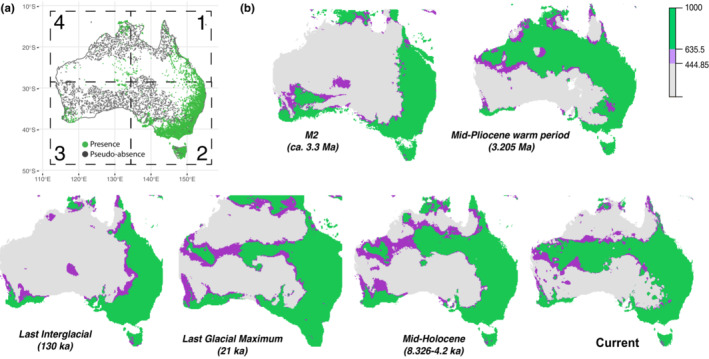
(a) Presence–absence points of *Trichosurus vulpecula* in Australia used for environmental niche modeling, with presence records from the GBIF database and pseudo‐absence generated using the biomod2 SRE algorithm. Numbered squares represent the spatial blocks defined by blockCV and run separately to be combined in the ensemble modeling. (b) Weighted mean ensemble ecological niche modeling projections on current and past climate data (Mid‐Holocene, Last Glacial Maximum, Last Interglacial period, Mid‐Pliocene warm period, and Pliocene M2). Pixel of colors represents ensemble modeling scores; green, scores above the cut‐off maximizing sensitivity (635.5); purple, scores >70% of this cut‐off value (444.85); and gray, low scores signifying low probability of presence.

Although *Trichosurus vulpecula* occurs widely in Australia, population density is uneven; there are large areas in the west and south of the country where the species is not recorded (Figure 2a) and this could help explain lineage/subspecies distinctions. Lack of gene flow between brushtail possum populations in different habitats would allow fixing of local adaptations (Mallet, 2008). We predicted that past climate variation resulted in range shifts among brushtail possum populations across the Australian continent that resulted in a connection of habitat and populations that are now isolated. Using modern records of brushtail possum occurrence, we infer the environmental envelope of *Trichosurus vulpecula* and extrapolate past distributions based on models of past climate. We then use independent time‐calibrated phylogenetic analysis to test for the expected correlation between the timing of niche fragmentation and lineage splitting using a lineage‐specific rate of molecular evolution based on a fossil‐calibrated analysis.

## 2 METHODS

Six subspecies of brushtail possum *Trichosurus vulpecula* are currently recognized (Kerle et al., 1991), of which five are represented in our data: langkurr *T. v. arnhemensis*; koomal *T. v. hypoleucus*; bilda *T. v. vulpecula*; *T. v. johnstonii*; and *T. v. fuliginosus* (Figure 1). Only *T. v. eburacensis* from Cape York is unsampled here.

### 2.1 Niche modeling

To model the environmental envelope of *Trichosurus vulpecula*, we obtained occurrence data for the species in Australia from the Atlas of Living Australia (ala.org.au) which provided us with data from 80 different datasets (Appendix S1) including museum records from regions where they are not present anymore (dating as early as 1857; Kerle, 2002). Filtering duplicate records at a 0.1° latitude and longitude resolution resulted in a dataset of 6297 records. Niche modeling approaches typically require both presence and absence data, but true absence information is difficult to verify. In this analysis, we used an alternative to true absence records by generating pseudo‐absence data using the surface range envelop algorithm (SRE) from R package “Biomod2” (Thuiller et al., 2015). A total of 3000 pseudo‐absence points were generated in locations that have contrasting environmental conditions to the presence locations (Figure 2a). Defining and projecting the climatic envelope of *Trichosurus vulpecula* required current and past climatic data (Current, Mid‐Holocene, Last Glacial Maximum, Last Interglacial period, Mid‐Pliocene warm period, and Pliocene M2) for Australia and New Zealand (where translocated brushtail possums are thriving), and these were obtained from the Worldclim and Paleoclim databases (Brown et al., 2018; Fick & Hijmans, 2017). Multivariate environmental similarity surfaces analysis (MESS) was computed using the “dismo” R package (Hijmans et al., 2017) to check for the presence of non‐analogous conditions in all scenarios of projection and to select only appropriate predictors that would not require models to extrapolate into novel climates. Multicollinearity of predictors was assessed using variance inflation factors (VIF, Zuur et al., 2010), retaining predictors with VIF smaller than 10 using “usdm” R package (Naimi et al., 2014). To investigate potential local differences and encompass Australia's broad climatic diversity and subspecies delimitations, the presence–absence data were separated into four spatial blocks using the R package “BlockCV” (Valavi et al., 2019; Figure 2a, *T. v. fuliginosus* and *T. v. vulpecula* were integrated in the same block, and *T. v. johnsonii* with *T. v. eburacensis*). The four spatial blocks were iteratively removed from the ecological niche models. We used a genetic algorithm from the R package “SDMtune” (Vignali et al., 2020) to optimize the models’ hyperparameter configuration and obtain more robust models (Gradient Boosting Machine (GBM), Random Forest (RF), Artificial Neural Network (ANN), and Maximum Entropy (Maxent); Appendix S1: Table S1). The models were then computed using “Biomod2” in R (Thuiller et al., 2016) with 80% of the input data used to calibrate the models, with the remaining 20% used to test them. Presences were weighted inversely proportional to the sampling abundance in order to give more importance to under‐sampled areas. VarImport was set to 3, allowing three permutations to estimate variable importance. Each model was run on each of the four different blocks four times, and the model accuracy of each run was assessed using receiver operating characteristic (ROC) and true skill statistic (TSS). An ensemble model was then generated from the qualifying subset of runs with ROC score > 0.80. The importance of each variable was calculated to assess the main drivers (or their proxies) of the alternative models and response curves were plotted to visualize the effect of the variables on the predicted habitat suitability. Projections of the ensemble model were then computed on current and past climate conditions using ensemble mean weights model (EMmw); cut‐off values were determined by maximizing sensitivity (the proportion of observed presence correctly predicted) and specificity (the proportion of observed pseudo‐absence correctly predicted) of the model. Range change statistics were computed using the R package “SDMTools” (VanDerWal et al., 2014).

### 2.2 Acquisition of DNA sequence data

Tissue samples of *Trichosurus vulpecula* were provided by the Australian National Wildlife at Canberra (ANWC) and the Australian Biological Tissue Collection (ABTC) at South Australia Museum. New Zealand samples were donated from pest control trapping in the Manawatu region, North Island (Turitea), and added to this study in order to increase the sample size of the population from Tasmania and Victoria, which is their origin (Pracy, 1974). Total genomic DNA was extracted from 106 specimens using the GeneAid Tissue DNA Isolation Kit following the manufacturers' guidelines (Figure 1), and quantity and quality checked using PerkinElmer LabChip® GX Touch HT.

We examined the genealogy and population diversity of *Trichosurus vulpecula* across Australia with mtDNA D‐loop sequences (Neaves et al., 2016; Umbrello et al., 2020; Figure 1). Polymerase chain reaction (PCR) amplification targeted a 730 bp fragment of the mtDNA D‐loop with PCR primers Tcan_218f and Tvul_1023r designed for *Trichosurus*, using conditions described previously (Pattabiraman et al., 2022). Amplification products were sequenced using BigDye® chemistry (Perkin Elmer) on an ABI3730 DNA analyzer. Sequences were edited and aligned using the software Geneious Prime (v. 2021.1.1, BioMatters; Kearse et al., 2012).

Whole genomic DNA from two short‐eared possums (*Trichosurus caninus*) and a representative subsample of 17 *Trichosurus vulpecula* (Figure 1, Appendix S2: Table S3) specimens were selected for shotgun sequencing to provide raw data for assembly of whole mitochondrial genomes. High‐throughput DNA sequencing on the BGI DNBSEQ‐G50 platform was used to generate up to 100 million 100 bp paired‐end reads per sample. These were paired and mapped to a reference mitochondrial genome (GenBank ID: NC_003039, Phillips et al., 2001) in Geneious Prime (v. 2021.1.1, BioMatters; Kearse et al., 2012), using the Geneious internal mapper and at least five iterations. Once a draft had been assembled, the data were remapped to the consensus sequence and the result was annotated by comparison to a reference and independently via the MITOS webservice (Bernt et al., 2013), and verified by translation of putative coding regions. These mitochondrial sequences were combined to produce an alignment of 19 DNA sequences of 15,462 bp (GenBank accessions ON399553‐ON399584, see Appendix S2: Table S3). D‐loop sequences from the 17 *Trichosurus vulpecula* were extracted from the mitogenomes and included in the following analysis using this marker.

### 2.3 Population statistics

For visualization of evolutionary relationships among the sequence variants of D‐loop, median‐joining and minimum spanning network (Bandelt et al., 1999; Kruskal, 1956) were inferred using Popart (Leigh & Bryant, 2015) and R package “pegas” (Paradis, 2010).

Discriminant analysis of principle components (DAPC: Jombart et al., 2010) was perform on the D‐loop alignment using the “adegenet” package in R (Jombart, 2008) in order to describe the different population clusters. DAPC uses principal component analysis to select the components holding the most variance followed and then applies discriminant analysis that navigates among this variability to provide the clearest distinction between groups. We retained components that defined a total of 90% of the variance and we used the subspecies as the prior grouping factor for the analysis.

### 2.4 Phylogenetic analysis

The newly assembled mtDNA genomes from 17 *Trichosurus vulpecula* were aligned with two mtDNA genomes from *T. caninus*. Phylogenetic relationships among these were inferred using maximum‐likelihood and Bayesian inference methods using Raxml 8.2.12 (Stamatakis, 2014) and Mrbayes v3.2.7 (Ronquist & Huelsenbeck, 2003). First, the data were divided into four partitions separating RNA (12S, 16S, and tRNA) and each codon position of protein‐coding genes. Partitionfinder v2.1.1 (Lanfear et al., 2017) was then used to select the best‐fitting substitution model for the data for each partition under corrected Akaike information criterion (AICc). This analysis determined the best substitution models to be GTR + G for all partitions and we used Raxml to compute the maximum‐likelihood analyses with the default tree search approach using a simultaneous nearest‐neighbor interchange method and a neighbor‐joining tree as starting tree to estimate the ML tree topologies with 1000 bootstrap replicates (Felsenstein, 1985; Felsenstein & Kishino, 1993; Huelsenbeck & Hillis, 1993). Bayesian inference analyses were performed using unlinked parameters for each partition and allowing branch lengths to vary proportionately across partitions. The analysis consisted of two independent runs each with two simultaneous Markov chain Monte Carlo (MCMC) chains of 30 million generations sampled every 1000 generations. Convergence and stationarity of runs and burn‐in period (first 25%) were determined using Tracer v1.7 (Rambaut et al., 2015).

### 2.5 Time calibration

We created an alignment of the mitochondrial genome from 14 species representing Dasyuromorphia and Diprotodontia, the two biggest Australian marsupials' orders (NCBI GenBank; Appendix S2: Table S5), and including both *Trichosurus vulpecula* and *T. caninus*. A molecular dating analysis was performed with this alignment to generate branch rate estimates that could be applied to the dating analysis of intraspecific *Trichosurus* lineages. The data were partitioned into tRNA and rRNA, D‐loop, CDS first and second codon positions, and CDS third codon positions using Beauti (Drummond et al., 2012) to input the priors and parameters for the Beast model. Substitution models for each partition were determined using the “BeastModel” algorithm. Model parameters included an optimized relaxed clock to allow the substitution rates to vary among the different species. A Yule model was implemented as it is appropriate for a phylogeny of mammal species as it assumes lineages split (constant speciation rate) without extinctions (Reid & Carstens, 2012; Steel & McKenzie, 2001). For time calibration of this phylogeny, we added priors estimated in a previous analyses of marsupial evolution using fossil calibrations (Meredith et al., 2009). That analysis of a DNA sequence from five nuclear genes (protein‐coding portions of ApoB, BRCA1, IRBP, Rag1, and vWF) representing nine placental lineages and 22 marsupials included 32 hard time constraints based on both the fossil record and previous phylogenetic analyses (Meredith et al., 2008).

We applied mean times and 95% highest posterior density (HDP; to accommodate error around past estimates) to the nodes in our analysis corresponding to the *Austradelphia*, *Macropodiformes* + *Phalangeroidea*, and *Phalangeridae* lineages with corresponding normal probability distributions, 63.0 Ma (55.6–70.0), 45.3 Ma (39.0–52.0), and 17.4 Ma (13.6–21.7), respectively. The Markov chain Monte Carlo (MCMC) chain length was set to 100 million generations and sampled every 1000 generations and the model was then run through Beast v2.6.5 (Drummond et al., 2012). Convergence of chains and ESS of the Beast run were checked using Tracer (Rambaut et al., 2015), using the ESS score (>200) to assess that all the parameters properly converged. This provided node calibration for the split between *Trichosurus vulpecula* and *T. caninus* in a similar analysis of data representing 17 *T. vulpecula* individuals and 2 *T. caninus*. For this, we implemented a coalescent constant population model in Beast with an optimized relaxed clock to determine the mutation rate of each partition in the species', and a strict clock was then set to produce the final dated phylogeny. We used Treeannotator (Helfrich et al., 2019) to compile the trees from the Beast runs into a maximum clade credibility tree using a standard burn‐in of the first 10% of trees and median node heights. Densitree and Figtree were used to visualize trees (Bouckaert, 2010).

## 3 RESULTS

### 3.1 Ecological niche modeling

After filtering presence data, we had 5757 locality records of brushtail possums across Australia (Figure 2a). Mapping these records revealed five regions where living possums are documented in Australia, separated by areas of apparently uninhabited land (or sea). We retained five bioclimatic variables (following MESS and VIF analyses) that could be used over the region of study and over the four regions and six climatic conditions. Other bioclimatic variables were discarded to avoid extrapolating the models to never explored values and to improve robustness of models and projections (Appendix S1: Figure S1). Of the 64 runs, 55 had a ROC score greater than 0.80 and were retained for ensemble modeling (Appendix S1: Table S2). The ensemble model showed a good fit to the current range of *Trichosurus vulpecula*, with a cut‐off value of 635.5 that maximized specificity (>98%) but still had a very good sensitivity of >93%. Plotting the projected ENM used this cut‐off value (635.5) and 70% of this value to represent the available niche space (Figure 2b). The modeled potential distribution (where suitable climate currently exists) closely matched the current occupancy of brushtail possums with the addition of potentially suitable niche in Central‐west and Central‐east Australia where only a few presences were recorded, mainly corresponding to museum samples. In general, it appears that the current distribution of brushtail possums is probably constrained by the climate variables or their proxies in our models. Estimates of the importance of each of the climatic variables included in the model and their response curves revealed that the most influential parameters were precipitation of the driest month and precipitation of the warmest quarter in the MaxEnt model and precipitation of the wettest month for the other models (GBM, ANN, and RF) or their proxies (Appendix S1: Figures S1 and S2).

Projections of ENM on models of past climatic conditions suggest wider potential brushtail possum distribution during the LGM (Figure 2b). An expanded distribution would have facilitated gene flow between populations. Suitable habitat for brushtail possums probably expanded during cold glacial periods (Appendix S1: Figure S1) and retracted during warm interglacials. The implied expansion during the LGM of suitable niche space corresponds to increased land surface as a result of lower sea levels and would have increased the opportunity for connection between previously isolated populations. Our niche modeling suggests habitat connection during the LGM between Tasmanian forests and Southern Australia, and between available niche space in Northern and Eastern Australia. However, the habitat of *T. v. hypoleucus* in Southwestern Australia remained isolated from all other suitable regions during the LGM (Figure 2b). We suggest that the availability of habitat would have been similar during all previous climate oscillations of the late Pleistocene (interglacial/glacial cycle), limiting contact between eastern and western populations. Our Mid‐Holocene and Last Interglacial projections infer a similar distribution of suitable niche space to current conditions. The projection of our possum ENM onto the climate model for the Mid‐Pliocene warm period reveals potential niche space in Northern Territory Central Australia and north of Western Australia, contiguous with potential habitat in Eastern Australia (Figure 2b). In contrast, the projection of our possum ENM onto the climate model for 3.3 million years ago (Pliocene M2 period) suggests a wider availability of potential habitat in the southeast and a possible habitat connection between eastern and western niche space on the south coast.

### 3.2 Haplotypic diversity

**FIGURE 3:**
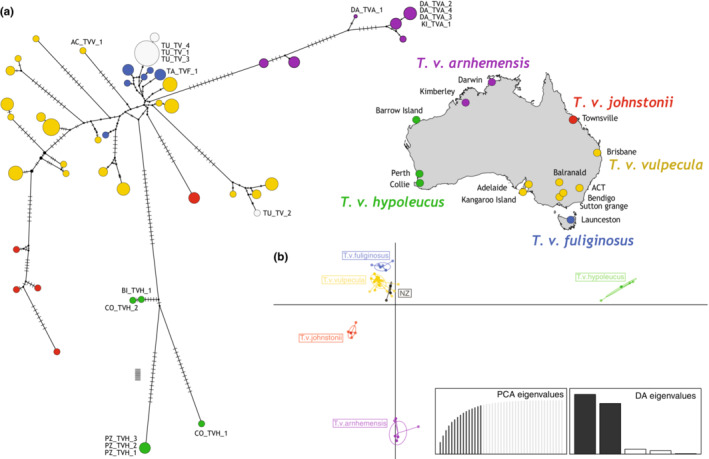
(a) Resolved minimum spanning haplotype network of mitochondrial D‐loop sequences of *Trichosurus vulpecula* subspecies from Australia and New Zealand. Samples used for phylogenetic and molecular clock analysis are indicated with specimen codes. Network nodes are scaled by number of individuals sharing a haplotype and colored by population origin. Number of steps between haplotypes is represented by dashes on the branches. (b) Scatterplot of the two main components of the DAPC analysis of D‐loop alignment from *Trichosurus vulpecula* in Australia and New Zealand with subspecies as prior grouping. Prior principal components analysis and discriminant analysis eigenvalues and selected components are also displayed. Each dot is an individual place with two coordinates on the discriminant analysis of two main components. The discriminant analysis uses genetic data to separate groups, and distance between groups on the discriminant analysis components correlates with the genetic distance of the groups.

Aligned D‐loop (Appendix S2: Table S4) sequence data comprised 561 base pairs for 106 specimens and revealed 44 haplotypes differing from one another by up to 7% (uncorrected). The haplotype network revealed clusters broadly concordant with sampling locations (Figure 3a). These clusters also appear in the DAPC analysis (Figure 3b) and correlate with the taxa *T. v. hypoleucus*, *T. v. arnhemensis*, and *T. v. johnstonii* and clustering of the southeastern samples (*T. v. vulpecula* and *T. v. fuliginosus*). The largest mtDNA distances were between *T. v. arnhemensis* in the north and *T. v. hypoleucus* in the southwest. The Tasmanian (*T. v. fuliginosus*) haplotypes nest among the haplotype diversity from New South Wales, Victoria, and South Australia *T. v. vulpecula* but are not shared. As expected, the haplotypes obtained from New Zealand brushtail possums are most similar to those of *T. v. fuliginosus* and *T. v. vulpecula*, but were unique within our sampling.

### 3.3 Whole mitochondrial sequences

The mitochondrial genome of *Trichosurus vulpecula* was 14,701 bp, containing the expected 37 genes (13 protein‐coding, 2 rRNA, and 22 tRNA). We aligned the 17 whole mtDNA sequences with two assembled from the related possum species *Trichosurus caninus* (Figure 1). Our phylogenetic analyses resolved the same strongly supported topology (Appendix S2: Figure S4) with three well‐supported lineages within our sampling of brushtail possums (maximum likelihood bootstrap score > 99 and Bayesian posterior probabilities >0.99). Samples from Southwest Australia (Perth and Collie) and Barrow Island that represent the subspecies *T. v. hypoleucus* form a lineage sister to the rest of the mtDNA diversity. Possums from Kimberley and Darwin in Northern Australia (*T. v. arnhemensis*) are sister to those from Victoria, Tasmania, and New Zealand (*T. v. fuliginosus* and *T. v. vulpecula*).

### 3.4 Molecular dating

**FIGURE 4:**
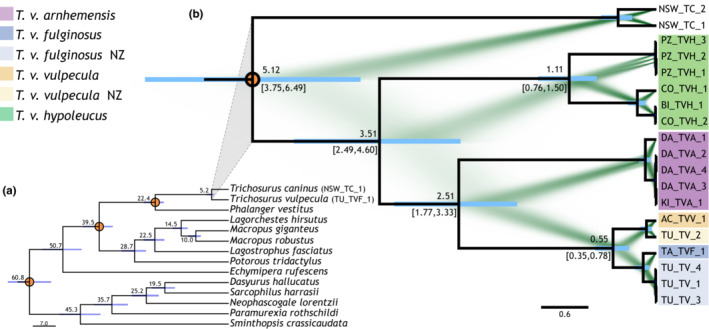
(a) Molecular clock‐calibrated phylogeny to estimate the most recent common ancestor of *Trichosurus vulpecula* and the short‐eared possum *T. caninus* using whole mitochondrial genomes. Calibration nodes (orange circles) based on dates from fossil‐calibrated analyses (Meredith et al., 2009). Maximum clade credibility tree using calibrated Beast analysis under Yule model, nodes corresponding to the divergence of lineages are dated in million years, and the bars represent the 95% HDP interval. (b) Phylogenetic hypothesis for brushtail possum *Trichosurus vulpecula* using whole mitochondrial genomes. Maximum clade credibility tree using calibrated Beast analysis under coalescent model overlapped with a representation of the different trees from the MCMC chain made using DensiTree. The nodes corresponding to the divergence of lineages are dated in million years and the 95% HDP interval is indicated and represented by the bars. Names of the sampled subspecies are indicated, and possum codes are colored accordingly.

We calibrated a phylogenetic tree using 16 species of Australian marsupial under a Yule model and three calibration points (based on previous estimates derived from fossil‐calibrated analyses). The best substitution models for the analysis determined by the beastModel algorithm was TN93, for both calibrated trees. DNA substitution rates from the optimized molecular clock analyses in Beast yielded site rates of 1.44E‐03 (±1.8E‐07) for the RNA partition, 1.21E‐03 (±1.2E‐07) for the partition including the nucleotides 1 and 2 of each codon of the CDSs, and 1.26E‐02 (±7.9E‐06) for the partition including the third nucleotides of each codon of the CDSs. This analysis had a high ESS score, and the node dates were consistent with our calibration except for the Phalangeridae which appear slightly older than previously reported, on our calibrated phylogeny (22.44 Ma (19.42–25.53); Figure 4a). This analysis allowed us to estimate the most recent common ancestor of the *T. vulpecula* and *T. caninus* mitochondrial lineage lived about 5.22 million years ago with 95% highest posterior density ranging between 4.10 and 6.48 Ma (Figure 4a). We used this estimate of the most recent common ancestor (MRCA) for our second calibrated tree with the *T. vulpecula* lineages evolving under a coalescent model, thus allowing us to estimate the timing of the divergence of the intraspecific diversity sampled. DNA substitution rates from the optimized molecular clock analyses in Beast yielded site rates of 1.6E‐03 (±7.28E‐8) for the RNA partition, 1.93E‐03 (±2.02E‐07) for the partition including the first and second codons of the CDSs, and 1.45E‐02 (±2.2E‐06) for the partition including the third codon of the CDSs; these rates were then used with a strict clock for the final run. This analysis produced a high ESS score and showed consistency with the root calibration. *Trichosurus v. hypoleucus* from Southwest Australia is sister to all the *T. vulpecula* diversity we sampled. The most recent common ancestor of *T. v. hypoleucus* with the rest of *T. vulpecula* diversity existed around 3.5 Ma (95%HDP 2.5–4.6 Ma). More recently, the common ancestor between *T. v. arnhemensis* and *T. v. vulpecula* and *T. v. fuliginosus* lived around 2.5 Ma (95%HDP 1.77–3.33 Ma). Our estimate of the timing of the most recent common ancestor of the two sublineages sampled from *T. v. fuliginosus* and *T. v. vulpecula* is dated to 0.55 Ma (95%HDP 0.35–0.78 Ma; Figure 4b).

## 4 DISCUSSION

In sexually reproducing animals, the fixation of adaptive traits in a population depends on the strength of natural selection and degree of gene flow (Mallet, 2008; Neher et al., 2010). While population size, range, and gene flow can be influenced by the emergence of adaptive traits (Reznick & Ghalambor, 2001; Szücs et al., 2017), climate and other abiotic factors have a strong influence on population structure (Davis & Shaw, 2001; Hewitt, 2004) and thus speciation (Endler, 1977). As a result, patterns of biodiversity and population structure are intrinsically linked to changes in Earth's climate (Byrne, 2008; Koot et al., 2022; Nogués‐Bravo et al., 2018). Here, we tested the origin of intraspecific division in the Australian brushtail possum that experiences very different habitats across its spatial range. The signatures of Pliocene divergence have been observed in a number of widespread Australian animals (Macqueen et al., 2010; Oliver et al., 2017; Potter et al., 2012; Rowe et al., 2008). Here, we not only infer that Pliocene fragmentation has shaped the current brushtail possums' population structure but also suggest that Pleistocene glacial cycles have had an impact on their current distribution (Bryant & Fuller, 2014; Chapple et al., 2005).

Regional biota across the vast landscape of Australia have distinct characteristics such that Southwest Australia is a recognized biodiversity hotspot of global significance with endemic plants that account for at least 1.4% of the world's plant species (Myers et al., 2000; Rix et al., 2015), and forests of East Australia are also recognized as globally significant for their distinct biodiversity (Williams et al., 2011). Indeed, seven major ecoregions are recognized across the continent, each having distinct environmental attributes (Mittermeier, 2004), and a high level of endemism among plant and animal assemblages suggest protracted regional coevolution (Braithwaite, 1990; Firman et al., 2020; New, 2011; Porder, 2014). Despite the very wide environmental range of the brushtail possum and the likely influence of biotic interactions limiting their current distribution (Abbott, 2012), our ecological niche models for *Trichosurus vulpecula* had both high sensitivity and specificity. The model suggested that regions of Western and Central Australia may have been suitable for brushtail possums before European settlements (Abbott, 2012; Kerle, 2002). During the Last Glacial Maximum (LGM), precipitation was lower than today (Faith et al., 2017; Sniderman et al., 2019), but we inferred extension of available niche space for *T. vulpecula* due to the global reduction in sea level that exposed low elevation land around Australia (Figure 2b). The distribution of D‐loop haplotypes confirms that Tasmanian *T. v. fuliginosus* is nested within the mtDNA diversity of *T. v. vulpecula* across the Bass Strait in Southeast Australia. Bass Strait today has a maximum depth of about 70 m, and lowered global sea level of about 120 m (Bintanja et al., 2005; Clark et al., 2009) during glacial stages of the Pleistocene would have resulted in a land connection across (Worth et al., 2017). Our inferred niche model confirms that the climate was welcoming for *Trichosurus vulpecula* in Tasmania at the LGM (Figure 2b), and our calibrated phylogenetic analysis estimated a relatively recent common ancestor of lineages sampled from *T. v. fuliginosus* and *T. v. vulpecula* at 550,000 years ago. This implies gene flow between Tasmania and Southeastern Australian populations during earlier glacial periods (e.g., marine isotope stages 7 and 9), suggesting they had not diverged sufficiently to prevent gene flow. Successful mixing of these two subspecies in New Zealand supports the idea that the two lineages have been isolated for brief periods followed by repeated connections maintaining reproductive compatibility (Pattabiraman et al., 2022). Perhaps the Tasmanian lineage could be better considered isolation by distance, rather than a “vicariant isolation” (Burridge, 2012).

Our projected niche model suggests that the connection of potential possum habitat would have been incomplete during the Pleistocene. In particular, our models suggest little if any suitable habitat in South Australia, even near the south coast. This, coupled with the inhospitable arid environment that prevailed across Central Australia throughout the Pleistocene would have severely limited or prevented gene flow between the western population (*Trichosurus v. hypoleucus*) and those to the east and north of Australia. The inferred distribution of suitable habitat during the LGM and presumably previous glacial phases suggests that the intraspecific diversity of brushtail possums might have originated well before the Pleistocene. Our ecological niche models suggest that the initial fragmentation of *Trichosurus vulpecula* occurred during the Pliocene. Our molecular dating analysis using whole mitochondrial genome data suggests that the most recent common ancestor of the sampled populations of brushtail possum in Australia existed in the middle of the Pliocene (about 3.5 million years ago; 95% HDP interval 2.4, 4.6 Ma).

**FIGURE 5:**
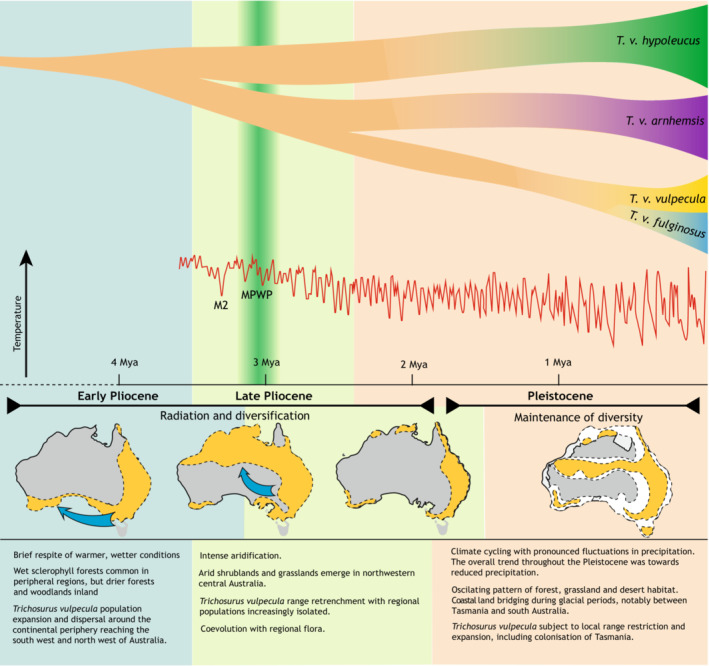
Hypothesis of intraspecific *Trichosurus vulpecula* differentiation in Australia inferred from calibrated phylogeny and niche modeling analyses. The main lineage splitting during the Pliocene corresponds to strong continental aridification, while Pleistocene glacial periods enabled exchange between Tasmania and South Australia due to lowered sea level. Aridification associated with the glacial period also restricted forest habitat that may have forced more intense plant–herbivore coevolution.

The phylogenetic results of this study would benefit from additional samples to include all six subspecies and samples from the Central and Eastern Australian populations. We have inferred phylogenies that are gene trees not subspecies trees, but there is no evidence that data from nuclear markers would change inferences about mtDNA lineage divergence. The concordant results from niche modeling and mtDNA phylogenetic analyses allow us to formulate an evolutionary scenario for brushtail possum in Australia (Figure 5). The divergence between *T. caninus* and *T. vulpecula* corresponds to approximately the end of the Miocene, a period that is marked by a dry and cold climate in Australia (Zachos et al., 2001). At the end of the Miocene, it is thought that rainforest persisted only in small patches in Southern and Southeastern Australia, with wet and open sclerophyll forests gradually replacing the rainforest (Black et al., 2012; Martin, 1998). This period of fragmented habitat might have been suitable for allopatric divergence of arboreal possums in their respective forest patches. Indeed, it appears that a number of modern marsupial groups (e.g., macropodine kangaroos, dasyurids, and peramelids) underwent extensive radiations during the Late Miocene, probably in response to this climate change (Beck, 2008; Meredith et al., 2009). Although late Miocene fossil deposits are rare in Australia, *Trichosurus* fossils have been found in the Riversleigh deposit (Archer et al., 2006; Roberts et al., 2008) confirming their presence in Eastern Australia at that time.

If the late Miocene can be considered a time of reduced niche space for Australian possums, then the early Pliocene provided more favorable conditions for brushtail possum population expansion. The short‐lived Pliocene reversal of aridification yielded warmer, wetter conditions across most of Australia (Sniderman et al., 2016). Pollen records show that rainforests as well as wet sclerophyll forests expanded in peripheral regions (Black et al., 2012; Martin, 2006; Mcgowran & Bock, 2000) including semiarid Southern Australia (Sniderman et al., 2016). Development of these types of habitats at this time plausibly enabled expansion of *T. vulpecula's* range around the drier heartland of Australia, so that they reached the west from the south and/or the north. Fossils of a related species, *Trichosurus hamiltonensis* (Flannery et al., 1987) found near Hamilton, Victoria, indicate that suitable habitat for brushtail possums was available there at that time.

The Late Pliocene saw increasingly dry conditions but much of Australia probably remained forested. The vegetation is thought to have consisted of sclerophyll open canopy forest but with arid shrublands and grasslands in Northwestern and Central Australia. This gradual change toward drier conditions and the expansion of arid ecosystems would very likely have reduced the available habitat for *T. vulpecula*. As habitat became more fragmented, brushtail possum populations would have become increasingly fragmented and experienced reduced gene flow, perhaps resulting in the current subspecies corresponding to the mtDNA lineage divergence we observe on our genetic data (Figures 3 and 5). Dating of most recent common ancestors observed within other Australian species such as the eastern dwarf tree frog *Litoria fallax* and blue‐tongued skink *Tiliqua rugosa* also corresponded to the Pliocene (Ansari et al., 2019; James & Moritz, 2000). By the end of the Pliocene, although wetter on average than today, the modern climate of Australia was largely established (Black et al., 2012; Martin, 2006).

Barrow Island population (Northwest Australia), which has been referred to as both *T. v. arhenemensis* (langkurr) and *T. v. hypoleucus* (koomal; Dunlop et al., 2021; Lynch et al., 2019), is interesting. Despite our ENM inference of little suitable habitat on the western coast of Australia, the molecular data suggest that the Barrow Island possums are part of the southwest mitochondrial lineage (koomal, *T. v. hypoleucus*). We do not know if the shared mtDNA lineage is due to human‐mediated translocation or natural gene flow between northwestern and southwestern populations before European settlement when their distribution was wider as suggested by historical records (Abbott, 2012). Inferences of wetter Early Pliocene conditions and fluctuating Pleistocene vegetation in this area (He & Wang, 2021; Sniderman et al., 2019), oceanic amelioration of coastal habitat, and periods of lowered sea level could plausibly combine to provide a dispersal corridor.

Overall, we find that fragmentation of populations during the Pliocene provides an explanation for the deep mitochondrial divergence within brushtail possums. Despite little phenotypic differentiation, *Trichosurus vulpecula* populations probably experienced little gene flow with one another since the Pliocene, supporting the recognition of several subspecies and explaining their local adaptations to wide range of climate zones they tolerate and the regional plant assemblages on which they feed.

## AUTHOR CONTRIBUTIONS

**David Carmelet‐Rescan:** Conceptualization (equal); data curation (equal); formal analysis (lead); investigation (lead); methodology (equal); software (lead); validation (equal); visualization (equal); writing – original draft (equal); writing – review and editing (equal). **Mary Morgan‐Richards:** Conceptualization (equal); supervision (equal); validation (equal); writing – original draft (equal); writing – review and editing (equal). **Nimeshika Pattabiraman:** Formal analysis (supporting); investigation (supporting); validation (equal). **Steven A. Trewick:** Conceptualization (equal); data curation (equal); project administration (equal); supervision (equal); validation (equal); visualization (equal); writing – original draft (equal); writing – review and editing (equal).

## FUNDING INFORMATION

Financial support for this research came from OSPRI New Zealand Ltd (Richard Curtis) and Predator Free 2050 Ltd (Dan Tompkins).

## CONFLICT OF INTEREST

The authors have no conflicts of interest to declare. All co‐authors have seen and agree with the contents of the manuscript and there is no financial interest to report. We certify that the submission is original work and is not under review at any other publication.

## Supporting information


Appendix S1.

## Data Availability

Sequence data used in this study are accessible on GenBank (accession numbers in the text and Supporting Information). Script and presence data of niche modeling analysis and intermediary files of the different phylogenetic analyses can be downloaded at https://doi.org/10.6084/m9.figshare.c.5975908.v2.
