# Supplementary material for: Time‐calibrated phylogeny and ecological niche models indicate Pliocene aridification drove intraspecific diversification of brushtail possums in Australia
Source: Ecol Evol. 2022 Dec 15;12(12):e9633. doi: 10.1002/ece3.9633 (PMC9755819; doi:10.1002/ece3.9633)

*Journal of Biogeography*

**Supporting information**

**Time-calibrated phylogeny and ecological niche models indicate Pliocene aridification drove intraspecific diversification of brushtail possums in Australia.**

David Carmelet-Rescan*, Nimeshika Pattabiraman, Mary Morgan-Richards, Steven A. Trewick

**Appendix S1** Niche modelling additional information

List of datasets used for presence: <https://doi.ala.org.au/doi/d542a045-55c0-4cd3-868f-83ebba172bbd>

**Table S1** MaxEnt **(a)**, Random forest **(b)**, ANN **(c)** and GBM **(d)** SDMTune results

**(a)**

| fc | reg | iter | train_AUC | test_AUC | diff_AUC |
| --- | --- | --- | --- | --- | --- |
| lqp | 2.6 | 130 | 0.95664297 | 0.9411 | 0.01554297 |
| lqp | 2.6 | 130 | 0.95664297 | 0.9411 | 0.01554297 |
| lqp | 2.6 | 130 | 0.95664297 | 0.9411 | 0.01554297 |
| lqp | 2.6 | 130 | 0.95664297 | 0.9411 | 0.01554297 |
| lqp | 2.6 | 130 | 0.95664297 | 0.9411 | 0.01554297 |
| lq | 2.6 | 130 | 0.95508359 | 0.93955 | 0.01553359 |
| lqp | 1.1 | 130 | 0.95085703 | 0.934575 | 0.01628203 |
| lqp | 0.9 | 130 | 0.94298984 | 0.925425 | 0.01756484 |
| lqph | 2.6 | 130 | 0.91173125 | 0.89985 | 0.01188125 |
| lqp | 0.9 | 290 | 0.82 | 0.8175 | 0.0025 |

**(b)**

| mtry | ntree | nodesize | train_AUC | test_AUC | diff_AUC |
| --- | --- | --- | --- | --- | --- |
| 3 | 500 | 1 | 0.99999922 | 0.9772625 | 0.02273672 |
| 3 | 600 | 1 | 0.99999922 | 0.977225 | 0.02277422 |
| 3 | 800 | 1 | 0.99999922 | 0.9771 | 0.02289922 |
| 3 | 600 | 1 | 0.99999922 | 0.977 | 0.02299922 |
| 3 | 600 | 1 | 0.99999922 | 0.976525 | 0.02347422 |
| 4 | 600 | 1 | 0.99999922 | 0.9763125 | 0.02368672 |
| 3 | 800 | 1 | 0.99999922 | 0.9760125 | 0.02398672 |
| 5 | 600 | 1 | 0.99999922 | 0.97595 | 0.02404922 |
| 3 | 600 | 1 | 0.99999922 | 0.975825 | 0.02417422 |
| 5 | 500 | 1 | 0.99999922 | 0.9757375 | 0.02426172 |

**(c)**

| mtry | ntree | nodesize | train_AUC | test_AUC | diff_AUC |
| --- | --- | --- | --- | --- | --- |
| 3 | 500 | 1 | 0.99999922 | 0.9772625 | 0.02273672 |
| 3 | 600 | 1 | 0.99999922 | 0.977225 | 0.02277422 |
| 3 | 800 | 1 | 0.99999922 | 0.9771 | 0.02289922 |
| 3 | 600 | 1 | 0.99999922 | 0.977 | 0.02299922 |
| 3 | 600 | 1 | 0.99999922 | 0.976525 | 0.02347422 |
| 4 | 600 | 1 | 0.99999922 | 0.9763125 | 0.02368672 |
| 3 | 800 | 1 | 0.99999922 | 0.9760125 | 0.02398672 |
| 5 | 600 | 1 | 0.99999922 | 0.97595 | 0.02404922 |
| 3 | 600 | 1 | 0.99999922 | 0.975825 | 0.02417422 |
| 5 | 500 | 1 | 0.99999922 | 0.9757375 | 0.02426172 |

**(d)**

| distribution | n.trees | interaction.depth | shrinkage | bag.fraction | train_AUC | test_AUC | diff_AUC |
| --- | --- | --- | --- | --- | --- | --- | --- |
| adaboost | 1800 | 7 | 0.028 | 0.2 | 0.99716484 | 0.98 | 0.01716484 |
| adaboost | 2400 | 4 | 0.028 | 0.2 | 0.99517734 | 0.979775 | 0.01540234 |
| adaboost | 2400 | 4 | 0.028 | 0.2 | 0.99527734 | 0.97955 | 0.01572734 |
| adaboost | 2400 | 4 | 0.028 | 0.2 | 0.99516484 | 0.979525 | 0.01563984 |
| adaboost | 2400 | 7 | 0.028 | 0.2 | 0.99873672 | 0.979475 | 0.01926172 |
| adaboost | 1800 | 7 | 0.028 | 0.2 | 0.99746328 | 0.978725 | 0.01873828 |
| adaboost | 1800 | 4 | 0.028 | 0.2 | 0.99354453 | 0.978475 | 0.01506953 |
| adaboost | 2400 | 4 | 0.028 | 0.2 | 0.99549141 | 0.97755 | 0.01794141 |
| adaboost | 2400 | 4 | 0.028 | 0.2 | 0.99573359 | 0.9768 | 0.01893359 |
| adaboost | 1800 | 4 | 0.028 | 0.2 | 0.99359297 | 0.976525 | 0.01706797 |

**Table S2** ROC score of each model for each run with the associated block. Run with a ROC score over 0.8 (in bold) were retained for the ensemble model.

| **Block** | **1** | **2** | **3** | **4** | **1** | **2** | **3** | **4** |
| --- | --- | --- | --- | --- | --- | --- | --- | --- |
| **Run** | **1** | **1** | **1** | **1** | **2** | **2** | **2** | **2** |
| RF | **0.864** | **0.963** | **0.924** | **0.966** | **0.864** | **0.963** | **0.924** | **0.966** |
| ANN | 0.702 | **0.909** | **0.925** | **0.935** | **0.903** | **0.902** | **0.899** | **0.943** |
| MAXENT | 0.592 | **0.891** | 0.770 | **0.939** | 0.592 | **0.891** | 0.770 | **0.939** |
| GBM | **0.888** | **0.955** | **0.890** | **0.971** | **0.888** | **0.955** | **0.890** | **0.971** |
| **Run** | 3 | 3 | 3 | 3 | 4 | 4 | 4 | 4 |
| RF | **0.864** | **0.963** | **0.924** | **0.966** | **0.864** | **0.963** | **0.924** | **0.966** |
| ANN | **0.887** | **0.911** | **0.925** | **0.954** | **0.885** | **0.935** | **0.890** | **0.925** |
| MAXENT | 0.592 | **0.891** | 0.770 | **0.939** | 0.592 | **0.891** | 0.770 | **0.939** |
| GBM | **0.888** | **0.955** | **0.890** | **0.971** | **0.888** | **0.955** | **0.890** | **0.971** |

**Figure S1** A) Result of MESS analysis for each set of bioclimatic variables for LGM, Mid-Holocene, Last Inter-Glacial period M2 and MPWP.


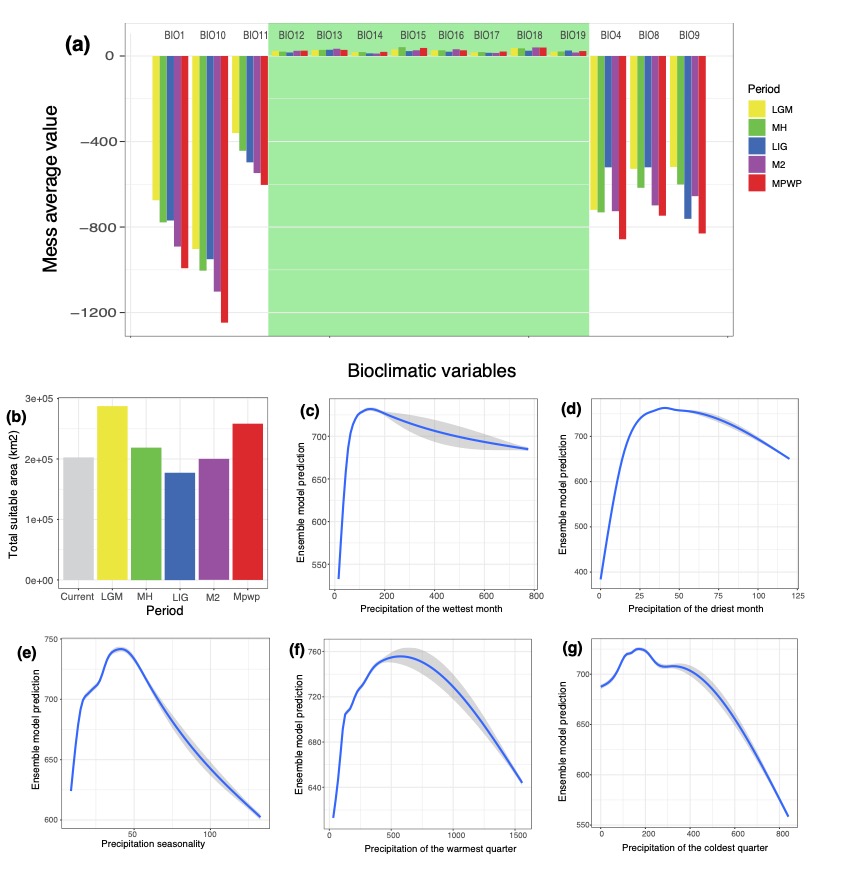


**Figure S2** Variable importance from the ENM.

**Appendix S2** Molecular analyses supplementary information

**Table S3**: Individual *Trichosurus* used for the different phylogenetic analyses with their associated location name, longitude, latitude and species and subspecies name. Australian samples came from the Australian National Wildlife Collection ANWC, and the Australian Biological Tissue Collection ABTC.

| Sample Name | Location | Longitude | Latitude | | *Trichosurus*  SPECIES | *Trichosurus* *vulpecula*  Subspecies | *MitochondriOn*  GENBANK ACCESSION # | Australian sample ID | Sample codes |
| --- | --- | --- | --- | --- | --- | --- | --- | --- | --- |
| NSW_Tc_1 | New South Wales, Australia | 152.995 | -30.165 | *caninus* | | NA | ON400096 | ANWCM16324 | 1Tc1b |
| NSW_Tc_2 | New South Wales, Australia | 152.995 | -30.165 | *caninus* | | NA | ON400092 | ANWCM24178 | 2Tc2b |
| BI_TvH_1 | Barrow Island, Western Australia | 115.4 | -20.79 | *vulpecula* | | *hypoleucus* | ON400081 | ABTC62933 | 6WA12 |
| CO_TvH_1 | Collie, Western Australia | 116.15 | -33.35 | *vulpecula* | | *hypoleucus* | ON400079 | ABTC83707 | 7WA13 |
| CO_TvH_2 | Collie, Western Australia | 116.15 | -33.35 | *vulpecula* | | *hypoleucus* | ON400080 | ABTC56745 | 10WA4 |
| PZ_TvH_1 | Perth Zoo, Western Australia | 115.85 | -31.95 | *vulpecula* | | *hypoleucus* | ON400082 | ABTC18396 | 11WA7 |
| PZ_TvH_2 | Perth Zoo, Western Australia | 115.85 | -31.95 | *vulpecula* | | *hypoleucus* | ON400083 | ABTC18394 | Pos18394 |
| PZ_TvH_3 | Perth Zoo, Western Australia | 115.85 | -31.95 | *vulpecula* | | *hypoleucus* | ON400078 | ABTC18395 | Pos18395 |
| TU_Tv_1 | Turitea, New Zealand | 175.6535 | -40.415 | *vulpecula* | | *vulpecula/fulginosis* | ON400086 | NA | PosTuGr2 |
| TU_Tv_2 | Turitea, New Zealand | 175.6535 | -40.415 | *vulpecula* | | *vulpecula/fulginosis* | ON400087 | NA | 3Tur9b |
| TU_Tv_3 | Turitea, New Zealand | 175.6535 | -40.415 | *vulpecula* | | *vulpecula/fulginosis* | ON400085 | NA | 4Tur10b |
| TU_Tv_4 | Turitea, New Zealand | 175.6535 | -40.415 | *vulpecula* | | *vulpecula/fulginosis* | ON400088 | NA | 5Tur11 |
| Ac_TVV_1 | Australian Capital Territory | 149.1333 | -35.217 | *vulpecula* | | *vulpecula* | ON400084 | ANWCM28611 | Pos28611 |
| TA_TVF_1 | Launceston, Tasmania | 147.08 | -41.26 | *vulpecula* | | *fulginosus* | ON400091 | ABTC50745 | Pos56745 |
| DA_TVA_1 | Darwin, Northern Territory | 130.84 | -12.46 | *vulpecula* | | *arnhemensis* | ON400093 | ABTC50841 | POS_M1 |
| DA_TVA_2 | Darwin, Northern Territory | 130.84 | -12.46 | *vulpecula* | | *arnhemensis* | ON400095 | ABTC50842 | POS_Wa_6 |
| DA_TVA_3 | Darwin, Northern Territory | 130.84 | -12.46 | *vulpecula* | | *arnhemensis* | ON400089 | ABTC50839 | 8NT14 |
| DA_TVA_4 | Darwin, Northern Territory | 130.84 | -12.46 | *vulpecula* | | *arnhemensis* | ON400090 | ABTC50840 | 9NT15 |
| KI_TVA_1 | Kimberley,Western Australia | 125.066 | -16.983 | *vulpecula* | | *arnhemensis* | ON400094 | ABTC7828 | Pos7828 |

**Table S4** Table of samples information, the majority of the sample are coming from Australian National Wildlife Collection of Mammals (2015-01-08).

| **Reg Number** | **State** | **Location** | **Latitude** | **Longitude** | **Collector** | **Catalogue record** | **Genebank Accession #** | **Subspecies** |
| --- | --- | --- | --- | --- | --- | --- | --- | --- |
| 50839 DA_TVA_1 DA_TVA_2 DA_TVA_3 DA_TVA_4 50840 50841 50842 50844 50845 50846 50847 50848 | NT | Darwin | -12.381 | 130.987 | NA | 554685 554686 554687 554688 554690 554691 554692 554693 554694 | ON342631 ON342630 ON342628 ON342618 ON342629 ON342622 ON342621 ON342620 ON342619 ON342625 ON342624 ON342626 ON342627 | *arnhemensis* |
| KI_TVA_1 | WA | Kimberley | -16.983 | 125.066 | NA | NA | ON342623 | *arnhemensis* |
| 50750 50751 50752 50753 50754 50746 50747 50748 50749 TA_TVF_1 | Tas | Launceston | -41.433 | 147.133 | Coman, Brian | 554592 554593 554594 554595 554596 554588 554589 554590 554591 | ON342655 ON342677 ON342676 ON342653 ON342678 ON342680 ON342679 ON342654 ON342681 ON342644 | *fulginosus* |
| BI_TvH_1 | WA | Barrow Island | -20.790 | 115.400 | NA | NA | ON342722 | *hypoleucus* |
| CO_TvH_1 CO_TvH_2 | WA | Collie | -33.350 | 116.150 | NA | NA | ON342721 ON342723 | *hypoleucus* |
| PZ_TvH_1 PZ_TvH_2 PZ_TvH_3 | WA | Perth Zoo | -31.950 | 115.850 | NA | NA | ON342718 ON342719 ON342720 | *hypoleucus* |
| 50815 50825 50816 50817 50819 50823 50824 | Qld | Townsville | -19.215 | 146.771 | NA | 554661 554662 554663 554665 554669 554670 | ON342685 ON342686 ON342687 ON342688 ON342640 ON342642 | *johnstonii* |
| Ac_TVV_1 | ACT | Australian Capital Territory | -35.217 | 149.133 | NA | NA | ON342643 | *vulpecula* |
| 50850 50851 50852 50853 50855 50857 | NSW | Armidale | -30.442 | 151.608 | NA | 554696 554697 554698 554699 554701 554703 | ON342701 ON342703 ON342704 ON342702 ON342700 ON342682 | *vulpecula* |
| 50827 50828 50829 50830 50831 50833 50834 50835 | Qld | Moggill area  Brisbane | -27.593 | 152.863 | NA | 554673 554674 554675 554676 554677 554679 554680 554681 | ON342693 ON342694 ON342689 ON342708 ON342691 ON342690 ON342692 ON342632 | *vulpecula* |
| 50784 50785 50786 50787 50788 50789 50790 50791 50792 | SA | Kingscote,  Kangaroo  Island | -35.650 | 137.633 | Coman, Brian | 554626 554627 554628 554629 554630 554631 554632 554633 554634 | ON342713 ON342714 ON342709 ON342710 ON342716 ON342717 ON342711 ON342712 ON342715 | *vulpecula* |
| 73756 7829 7830 132145 132163 132202 27443 10734 | SA | Adelaide | -34.751 | 138.897 | NA | NA | ON342707 ON342699 ON342705 ON342698 ON342696 ON342706 ON342695 ON342697 | *vulpecula* |
| 50707 50708 50710 50711 50712 50713 50714 50715 50716 | Vic | Sutton Grange | -36.983 | 144.350 | Coman, Brian | 554542 554543 554546 554547 554548 554549 554550 554551 554552 | ON342657 ON342660 ON342659 ON342661 ON342649 ON342650 ON342651 ON342652 ON342648 | *vulpecula* |
| 50728 50729 50730 50731 50732 50733 50734 50735 50736 50737 | Vic | Tang Tang Swamp,  Bendigo | -36.367 | 144.300 | Coman, Brian | 554564 554565 554566 554567 554568 554569 554570 554571 554572 554573 | ON342636 ON342638 ON342683 ON342635 ON342634 ON342658 ON342684 ON342637 ON342656 ON342639 | *vulpecula* |
| TU_Tv_1 TU_Tv_2 TU_Tv_3 TU_Tv_4 | NZ | Turitea | -40.415 | 175.654 | NA | NA | ON342647 ON342645 ON342633 ON342646 | *vulpecula &*  *fulginosis* |
| Tvul_Tara_10 Tvul_Tara_12 Tvul_Tara_13 Tvul_Tara_15 Tvul_Tara_16 Tvul_Tara_17 Tvul_Tara_19 Tvul_Tara_20 Tvul_Tara_21 Tvul_Tara_22 Tvul_Tara_23 Tvul_Tara_24 Tvul_Tara_25 Tvul_Tara_26 | NZ | Taranaki | -39.166 | 173.960 | NA | NA | ON342672 ON342668 ON342674 ON342665 ON342667 ON342666 ON342662 ON342669 ON342663 ON342671 ON342673 ON342664 ON342670 ON342675 | *vulpecula &*  *fulginosis* |

**Table S5** Marsupial mtDNA genomes used for the molecular clock analysis with the species names, Genbank accession numbers and the associated publications.

| **Species name** | **Genbank accession number** | **Publication** |
| --- | --- | --- |
| *Macropus robustus* | NC_001794 | (Janke et al., 1997) |
| *Macropus giganteus* | KY996502 | (Nilsson et al., 2018) |
| *Lagorchestes hirsutus* | NC_008136 | (Munemasa et al., 2006) |
| *Lagostrophus fasciatus* | NC_008447 | (Nilsson, 2006) |
| *Potorous tridactylus* | NC_006524 | (Nilsson et al., 2004) |
| *Phalanger vestitus* | NC_008137 | (Munemasa et al., 2006) |
| *Sminthopsis crassicaudata* | AY795974 | (Phillips et al., 2006) |
| *Neophascogale lorentzi* | KJ868130 | (Mitchell et al., 2014) |
| *Dasyurus hallucatus* | AY795973 | (Phillips et al., 2006) |
| *Sarcophilus harrisii* | NC_018788 | (Miller et al., 2011) |
| *Paramurexia rothschildi* | KJ868134 | (Mitchell et al., 2014) |

Janke, A., Xu, X., & Arnason, U. (1997). The complete mitochondrial genome of the wallaroo (Macropus robustus) and the phylogenetic relationship among Monotremata, Marsupialia, and Eutheria. *Proceedings of the National Academy of Sciences of the United States of America*, *94*(4), 1276–1281. https://doi.org/10.1073/pnas.94.4.1276

Miller, W., Hayes, V. M., Ratan, A., Petersen, D. C., Wittekindt, N. E., Miller, J., Walenz, B., Knight, J., Qi, J., Zhao, F., Wang, Q., Bedoya-Reina, O. C., Katiyar, N., Tomsho, L. P., Kasson, L. M. C., Hardie, R. A., Woodbridge, P., Tindall, E. A., Bertelsen, M. F., … Schuster, S. C. (2011). Genetic diversity and population structure of the endangered marsupial Sarcophilus harrisii (Tasmanian devil). *Proceedings of the National Academy of Sciences of the United States of America*, *108*(30), 12348–12353. https://doi.org/10.1073/pnas.1102838108

Mitchell, K. J., Pratt, R. C., Watson, L. N., Gibb, G. C., Llamas, B., Kasper, M., Edson, J., Hopwood, B., Male, D., Armstrong, K. N., Meyer, M., Hofreiter, M., Austin, J., Donnellan, S. C., Lee, M. S. Y., Phillips, M. J., & Cooper, A. (2014). Molecular phylogeny, biogeography, and habitat preference evolution of Marsupials. *Molecular Biology and Evolution*, *31*(9), 2322–2330. https://doi.org/10.1093/molbev/msu176

Munemasa, M., Nikaido, M., Donnellan, S., Austin, C. C., Okada, N., & Hasegawa, M. (2006). Phylogenetic analysis of diprotodontian marsupials based on complete mitochondrial genomes. *Genes and Genetic Systems*, *81*(3), 181–191. https://doi.org/10.1266/ggs.81.181

Nilsson, M. A. (2006). Phylogenetic relationships of the Banded Hare wallaby (Lagostrophus fasciatus) and a map of the kangaroo mitochondrial control region. *Zoologica Scripta*, *35*(4), 387–393. https://doi.org/10.1111/j.1463-6409.2006.00237.x

Nilsson, M. A., Arnason, U., Spencer, P. B. S., & Janke, A. (2004). Marsupial relationships and a timeline for marsupial radiation in South Gondwana. *Gene*, *340*(2), 189–196. https://doi.org/10.1016/j.gene.2004.07.040

Nilsson, M. A., Zheng, Y., Kumar, V., Phillips, M. J., & Janke, A. (2018). Speciation generates mosaic genomes in Kangaroos. *Genome Biology and Evolution*, *10*(1), 33–44. https://doi.org/10.1093/gbe/evx245

Phillips, M., McLenachan, P., Down, C., Gibb, G., & Penny, D. (2006). Combined mitochondrial and nuclear DNA sequences resolve the interrelations of the major Australasian marsupial radiations. *Systematic Biology*, *55*(1), 122–137. https://doi.org/10.1080/10635150500481614

**Figure S4**: Phylogenetic tree of the different MtDNA lineages inferred using Bayesian inference methods (MrBayes) and maximum likelihood methods (RaXML). Numbers at nodes indicate posterior probabilities for the Bayesian inference analysis and bootstrap values for maximum likelihood analysis.


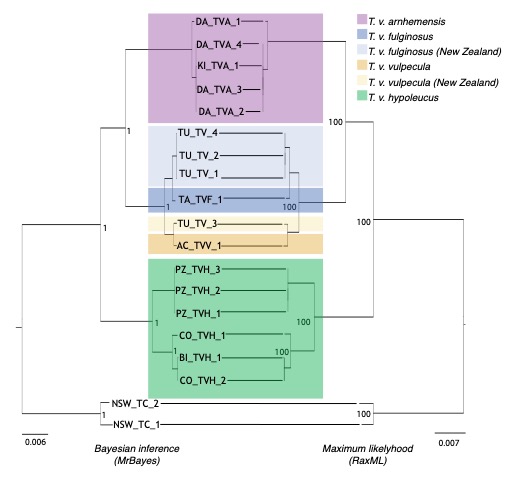

Supplement: Supplementary file 1 — Appendix S1. [file ECE3-12-e9633-s001.docx]
